# Supplementary material for: Organelle proteomics reveals novel metabolic vulnerabilities in FLT3-ITD cells
Source: Leukemia. 2026 Jun 9;40(8):1657–67. doi: 10.1038/s41375-026-03000-6 (PMC13421297; doi:10.1038/s41375-026-03000-6)
Supplement: Supplementary file 1 — Supplementary figures [file 41375_2026_3000_MOESM1_ESM.docx]

**Supplementary figures**


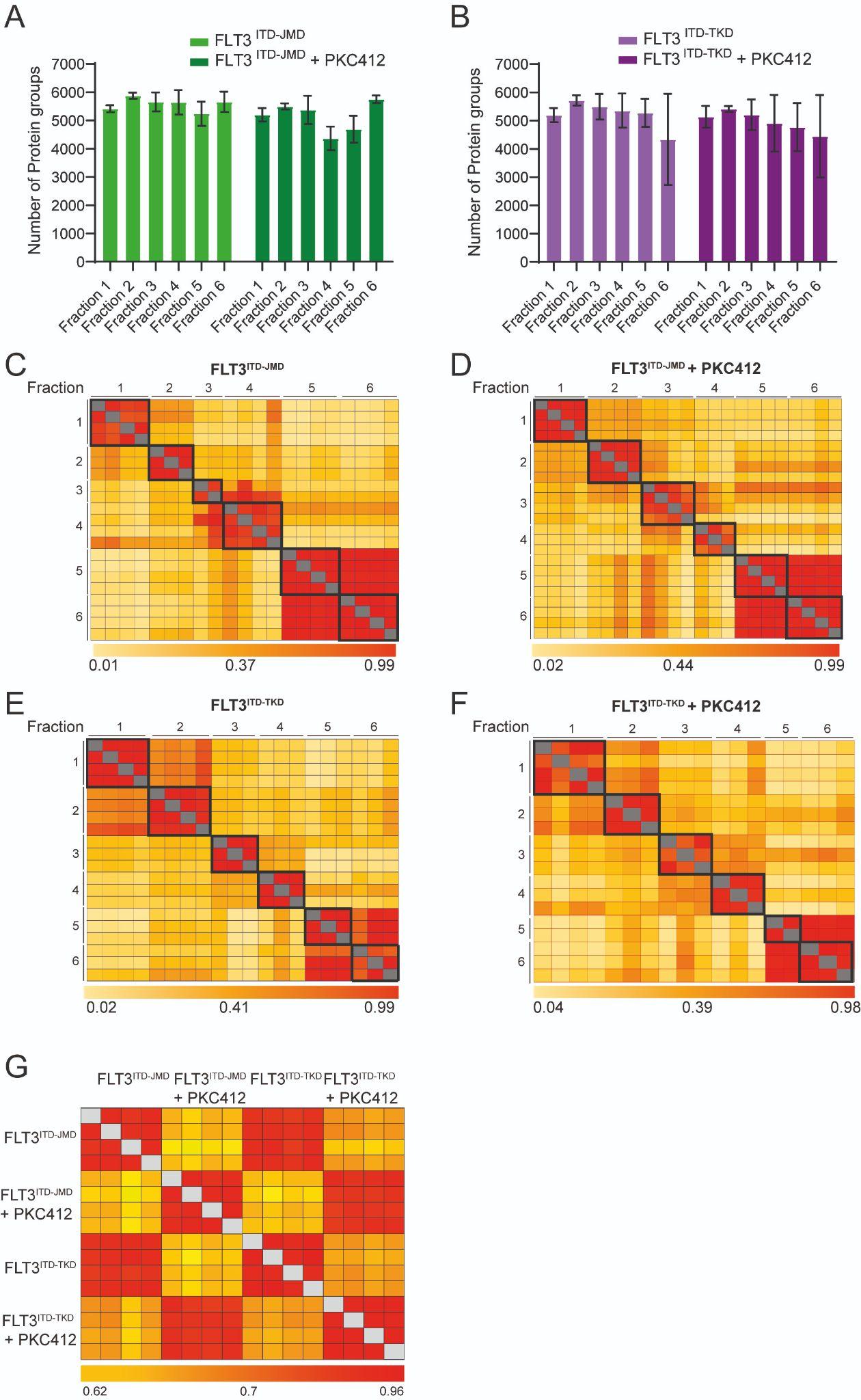


**Figure S1.** (**A-B**) Bar plots reporting the number of peptides identified in each biological condition and subcellular fraction in FLT3^ITD-JMD^ (A) and FLT3^ITD-TKD^ (B). (**C-F**) Heatmaps reporting the Pearson Correlation coefficient of the proteomic analysis of the subcellular fractionation of control FLT3^ITD-JMD^ (C), midostaurin-treated FLT3^ITD-JMD^ (D), FLT3^ITD-TKD^ (E) and midostaurin-treated FLT3^ITD-TKD^ (F). (**G**) Heatmap reporting the Pearson Correlation coefficient of the phosphoproteomic analysis of control and midostaurin-treated FLT3^ITD-JMD^ and FLT3^ITD-TKD^.

**
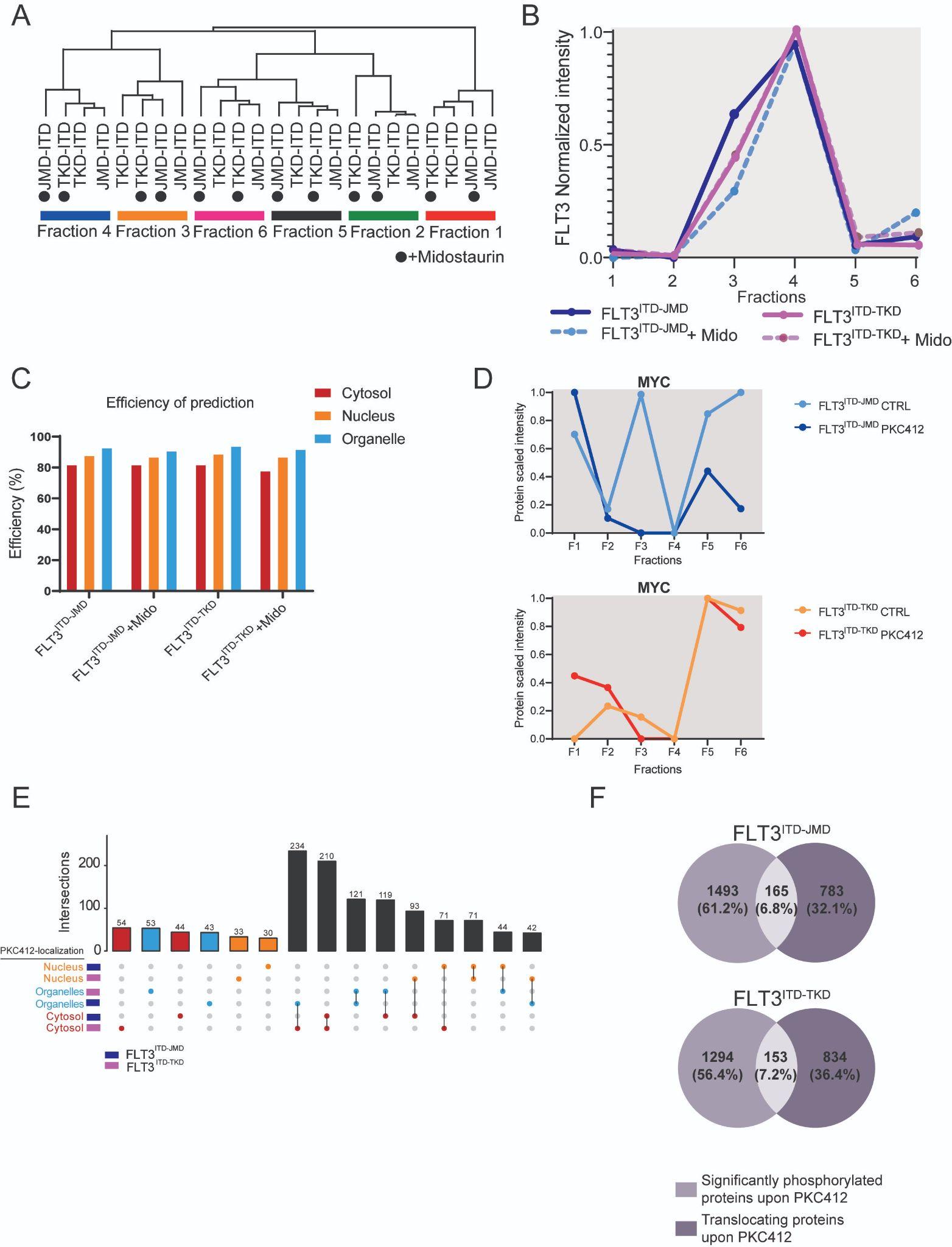
**

**Figure S2.** **(A)** Hierarchical clustering of subcellular fractionation data. **(B)** Intensity profiles of FLT3 protein in the six fractions (scaled intensities). **(C)** Bar plot reporting the percentage of the prediction efficiency of the SVM-organelle assignment. **(D)** Intensity profiles of MYC protein in the six fractions (scaled intensities). **(E)** Upset plot showing the number of translocating proteins that share the same subcellular location after midostaurin treatment in FLT3^ITD-JMD^ versus FLT3^ITD-TKD^ cells, or unique to each condition. **(F)** Venn diagrams reporting the percentage of translocating proteins whose phosphorylation is significantly modulated by midostaurin treatment or not.

**
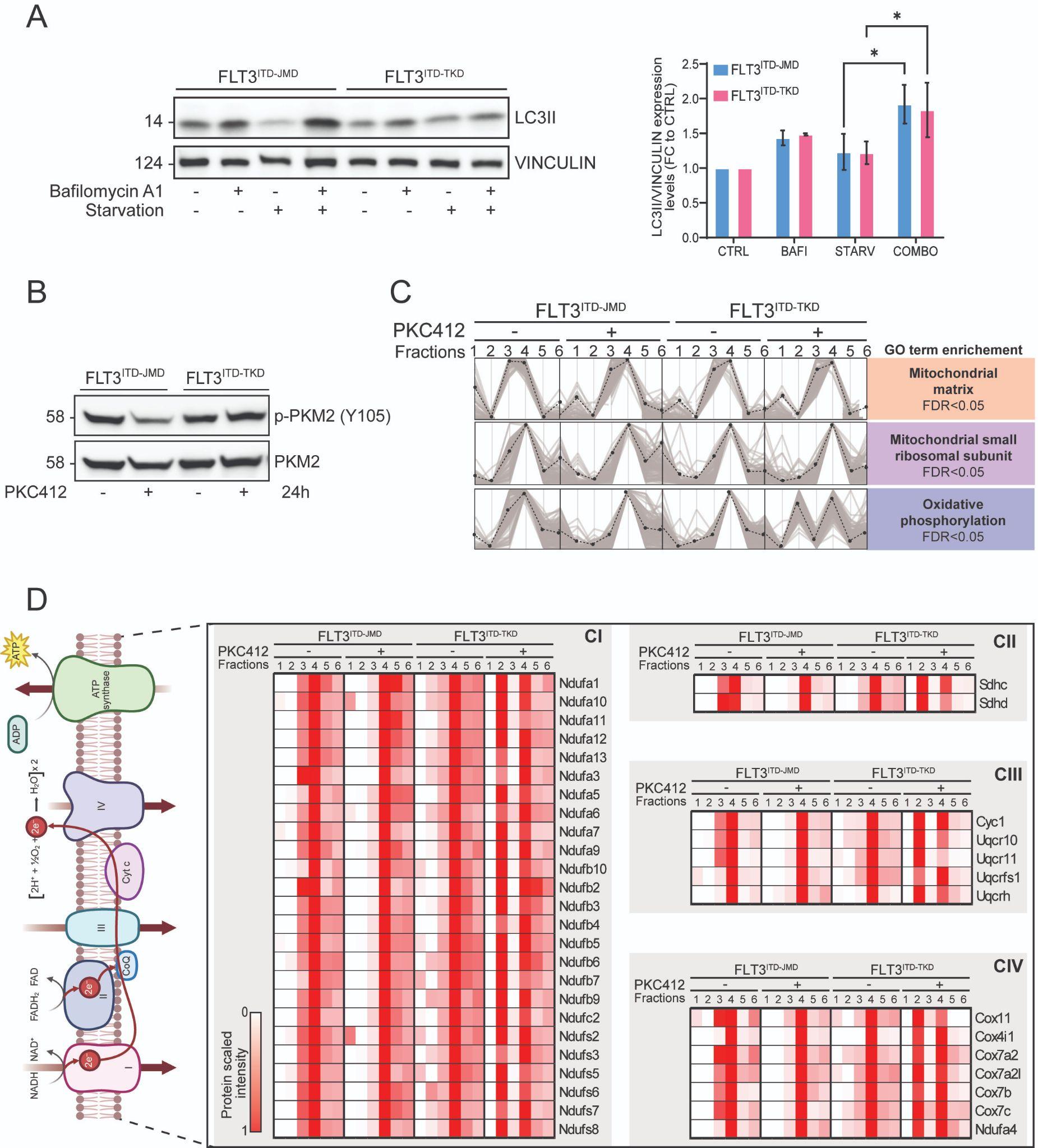
**

**Figure S3. (A)** Representative western blot and relative quantification of LC3-II protein levels after induction of autophagy by starvation in FLT3^ITD-JMD^ and FLT3^ITD-TKD^ either alone or combined with midostaurin treatment. (**B**) Representative western blot reporting the phosphorylation level of PKM2 at Y105 in FLT3^ITD-JMD^ and FLT3^ITD-TKD^ after 24 hours of 100nM midostaurin treatment. (**C**) Intensity profiles of the 168 proteins annotated in the MitoCarta database as mitochondrial proteins across the six fractions in each experimental condition. Colored boxes indicate the enriched GO-Term biological processes and cell compartments. (**D**) Heatmaps reporting the protein levels (scaled intensities) of the OXPHOS related proteins across the 6 fractions in each experimental condition.


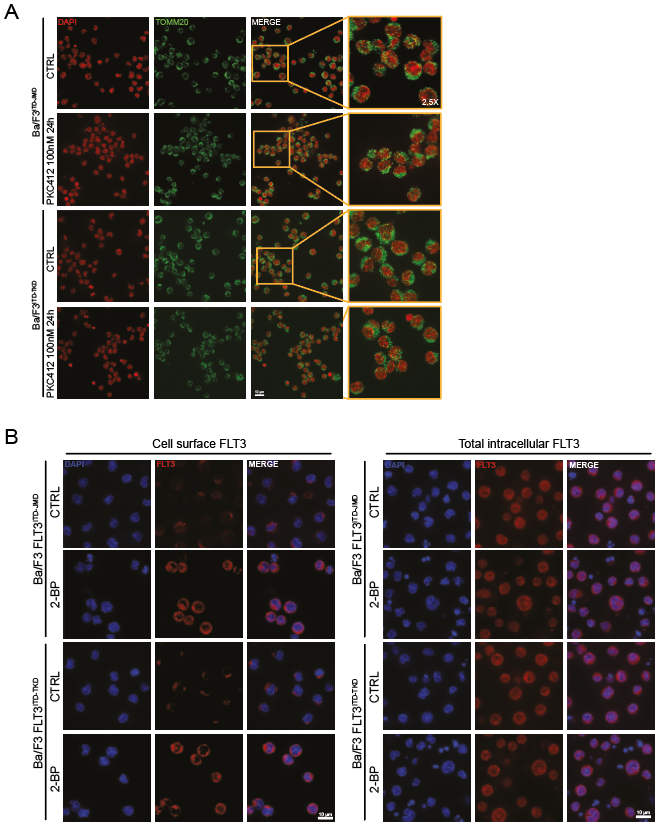


**Figure S4.** (**A**) Representative immunofluorescence images of FLT3^ITD-JMD^ and FLT3^ITD-TKD^ treated in control condition (CTRL) or upon midostaurin treatment (PKC412; 100 nM, 24h). Nuclei were stained with DAPI (red) and mitochondria were labelled with anti-TOMM20 antibody (green). (**B**) Representative immunofluorescence images of FLT3 protein (red) in unpermeabilized and permeabilized FLT3^ITD-JMD^ and FLT3^ITD-TKD^ expressing cells in control condition and upon 24 hours 50μM 2-bromopalmitate exposure.
