# Supplementary material for: Organelle proteomics reveals novel metabolic vulnerabilities in FLT3-ITD cells
Source: Leukemia. 2026 Jun 9;40(8):1657–67. doi: 10.1038/s41375-026-03000-6 (PMC13421297; doi:10.1038/s41375-026-03000-6)
Supplement: Supplementary file 2 — Supplementary material [file 41375_2026_3000_MOESM2_ESM.docx]

**Organelle proteomics reveals novel metabolic vulnerabilities in FLT3-ITD cells**

Valeria Bica^1,2*^, Anna Francesca Pacilè^1*^, Martin Boettcher^3,4^, Valentina Marano^2^, Veronica Marabitti^1^, Francesca Nazio^1^, Mirko Cortese^2^, Thomas Fischer^3,4^, Livia Perfetto^5^, Dimitrios Mougiakakos^3,4^, Giorgia Massacci^1#^, Francesca Sacco^1,2 #^.

**Cell culture**

Mouse Ba/F3 cells expressing ITD-JMD and ITD-TKD constructs were provided by courtesy of T. Fischer. To recapitulate functional properties driven by the differentially located FLT3-ITD-mutations, we used the following cell lines:

- ITD-JMD 598/599 (22 amino acids)
- ITD-TKD 612/613 (33 amino acids)


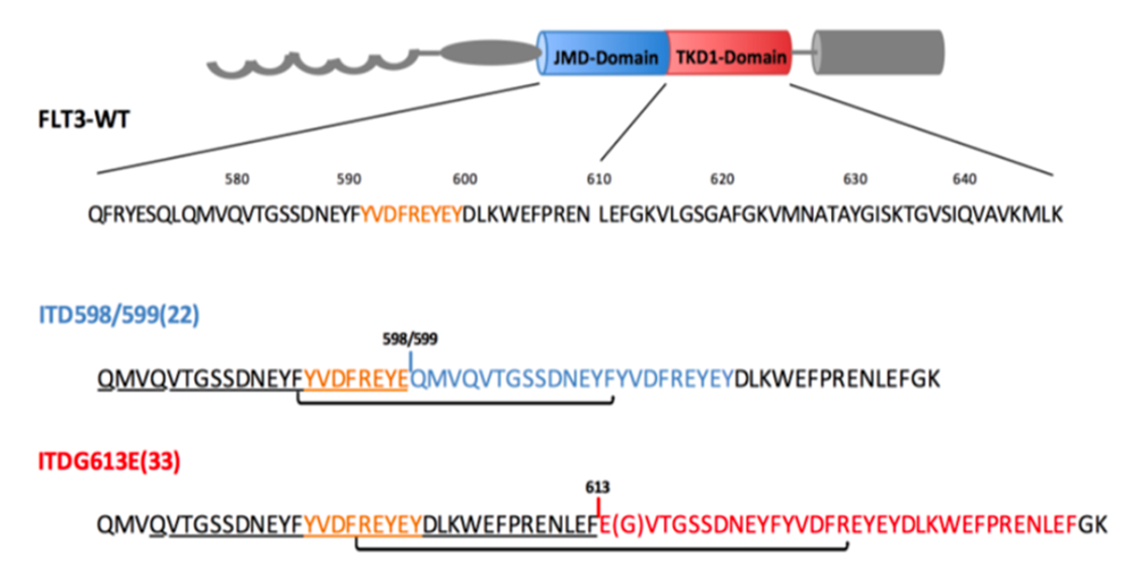


Cells were cultured in RPMI 1640 medium (Hyclone, Thermo Scientific, Waltham, MA) supplemented with 10% heat-inactivated fetal bovine serum (ECS0090D Euroclone, Italy, MI ), 100 U/ml penicillin and 100 mg/ml streptomycin (Gibco 15140122), 1 mM sodium pyruvate (Sigma-Aldrich, St. Louis, Missouri, United States, S8636) and 10 mM 4-(2-hydroxyethyl)-1-piperazineethanesulfonic acid (HEPES) (Sigma H0887). Midostaurin (Selleck chemical, S8064) was used at 100 nM for 24 hours. Cells were routinely tested for mycoplasm infection.

**Immunoblot analysis**

BaF3 cells were seeded at a concentration of 500.000 cells/ml and treated as indicated. After treatments cells were centrifuged and washed in PBS 1x.  Next, cells were lysed in ice-cold lysis buffer (150 mM NaCl, 50 mM Tris–HCl, pH 7.5, 1% Nonidet P-40, 1 mM EGTA, 5 mM MgCl_2_, and 0.1% SDS) supplemented with 1 mM PMSF, 1 mM ortovanadate, 1 mM NaF, protease inhibitor mixture 1×, inhibitor phosphatase mixture II 1×, and inhibitor phosphatase mixture III 1× and incubated for 30 min. Protein lysates were separated at 13,000*g* for 30 min. The total protein concentration was determined using the Bradford reagent (Biorad, 5000006). Protein extracts were denatured and heated at 95°C for 10 min in NuPAGE LDS Sample Buffer (Thermo Fisher Scientific, NP0007) and a buffer contained DTT as a reducing agent (NuPAGE Sample Reducing Agent) (Thermo Fisher Scientific, NP0004). Proteins were resolved using 4–15% Bio-Rad Mini-PROTEAN TGX/CRITERION polyacrylamide gels (Bio-Rad 4561084). Proteins were transferred to Trans-Blot Turbo Mini Nitrocellulose Membranes using a Trans-Blot Turbo Transfer System (Bio-Rad, 17001918), and the nonspecific binding membranes were saturated in blocking solution (5% skimmed milk powder, 0.1% Tween 20 in 1× TBS) at room temperature for 1 hour. Saturated membranes were incubated overnight with primary antibodies diluted in BSA 5%. HRP-conjugated secondary antibodies (Goat Anti-Mouse/Anti-Rabbit IgG (H+L)-HRP Conjugate 1:3000, BIORAD 1721011) were diluted in blocking solution and used for the detection of the primary antibodies. Chemiluminescence was detected using Clarity Western ECL Blotting Substrates (Bio-Rad) and the Las-3000 Imaging System (Fujifilm). Band densities were quantified using ImageJ and normalized to the loading control.

**Nutrient Starvation**

For glucose deprivation, cells were cultured in glucose-free RPMI 1640 medium (Thermo Fisher Scientific) supplemented with 10% dialyzed fetal bovine serum (FBS) , 2mM glutamine, 1% Hepes, and 1% penicillin-streptomycin for the indicated time points. For amino acid deprivation, cells were maintained in amino acid–free RPMI 1640 (Thermo Fisher Scientific) supplemented with 10% dialyzed FBS and 1% penicillin-streptomycin. For lipid deprivation, cells were cultured in RPMI 1640 containing, 1% penicillin-streptomycin and 10% lipid-depleted FBS, prepared as indicated following. Briefly, FBS was warmed to room temperature and fumed silica (hydrophilic) was added at 0.5g per 10 mL of serum. The mixture was incubated at 4°C overnight. Following incubation, the silica-lipid complexes were removed by centrifugation at 1500 × g for 20 minutes. The clarified supernatant was carefully collected and sterile filtered through a 0.22μm filter to remove any remaining particles. The resulting lipid-depleted FBS was aliquoted and stored at −20°C or −80°C until use. Control cells were cultured in RPMI 1640 with 10% standard FBS. After the starvation period, cells were processed for downstream assays.

**MTT assay**

Cell viability was measured using the Cell Proliferation Kit I (MTT) (Roche). Cells were treated as indicated. Then, MTT was added to the cells and incubated for 4 hours at 37 ◦C. Solubilization Solution was used to dissolve the formazan crystals during an overnight incubation. Finally, the plates were read at 590nm using a microplate reader (Bio-Rad).

**Immunofluorescence analysis**

Coverslips were incubated with poly-L-Lysine solution (Santa Cruz Biotechnology, sc-286689) for 1 hour at 37°C to allow cells’ adhesion. Upon treatment, cells were washed with 1x PBS and spotted on coverlips. Next, cells were fixed in 4% PFA for 15 min at RT and permeabilized with 0.3% Triton X-100 in 1x PBS for 10 min at RT. Unspecific bindings were saturated by incubating samples in blocking solution (3% BSA and 0.3% Triton X-100 in 1× PBS) for 1 hour. Cells were incubated with primary antibodies diluted in blocking solution according to manufacturer instruction. After incubation, cells were washed twice with 1x PBS and incubated, for 1h at RT, with host-specific secondary antibodies and 1:2000 DAPI solution (Thermo Scientific, 62248) diluted in blocking solution. Finally, the cells were washed twice with 1x PBS, mounted on slides and let dry overnight at RT.

**Subcellular fractionation**

Subcellular fractionation was performed following a recent in-house-developed spatial proteomics workflow (3) in biological quadriplicates. The workflow requires the preparation of three different lysis buffers:

| Buffer A | 30 mM Hepes pH 7.4; 15 mM NaCl, 2 mM MgCl2, 1 mM EDTA |
| --- | --- |
| Buffer B | 30 mM Hepes pH 7.4; 15 mM NaCl, 2 mM MgCl2, 1 mM EDTA, 350 mM sucrose |
| Buffer C | 30 mM Hepes pH 7.4; 15 mM NaCl, 2 mM MgCl2, 1 mM EDTA, 20% glycerol |

Protease and phosphatase inhibitors were added to each buffer at the final concentrations of: 1 mM PMSF, 1 mM ortovanadate, 1 mM NaF, protease inhibitor mixture 1×, inhibitor phosphatase mixture II 1×, and inhibitor phosphatase mixture III 1×.  n. Cell pellets were resuspended in 540 µl of Buffer B and 60 µl of 0.15% digitonin solution 5 min. Samples were incubated on orbital shaking at 4°C for 30 min and centrifuged for 3 min at 500 g. The recovered supernatant was marked as Fraction 1. Cell pellets were washed twice with 1 ml of buffer C. Cell pellets were resuspended in 540 µl of buffer B and 60 µl of 1.4 M NaCl. Samples were incubated on orbital shaking at 4°C for 30 min and centrifuged for 3 min at 500 g. The recovered supernatant was marked as Fraction 2. Cell pellets were washed twice with 1 ml of buffer B. Cell pellets were resuspended in 570 µl of buffer B and 30 µl of 10% Tween-20. Samples were incubated on orbital shaking at 4°C for 30 min and centrifuged for 3 min at 500 g. The recovered supernatant was marked as Fraction 3. Cell pellets were washed twice with 1 ml of buffer B. Cell pellets were resuspended in 540 µl of buffer C and 60 µl of 10% N-dodecyl maltoside. Samples were incubated on orbital shaking at 4°C for 30 min and centrifuged for 3 min at 500 g. The recovered supernatant was marked as Fraction 4. Cell pellets were washed twice with 500 µl of buffer C and resuspended in 540 µl of buffer A, 60 µl of 5 M NaCl and 1 µl of Benzonase® Nuclease. Samples were incubated on orbital shaking at 4°C for 30 min and centrifuged for 3 min at 500 g.  Supernatant was recovered and marked as Fraction 5. Cell pellets were washed once with 500 µl of buffer C and cell pellets were resuspended in 500 µl of buffer A 60 µl of 1.4 M NaCl and 18 µl of 10% SDS. Samples were immediately boiled for 10 min at 95 °C and marked as Fraction 6. Finally, all the fractions were centrifuged for 10 min at maximum speed.

**Samples preparation for proteomic analysis**

Protein precipitation in the 6 fractions was performed with methanol/chloroform precipitation and samples were resuspended in 2% SDC buffer in 100 mM Tris -HCl (pH 8.5). Proteins were reduced and alkylated with TCEP and CAA (1:100 v/v) at 45° for 5 minutes. Proteins digestion was performed adding trypsin and LysC enzymes (1:100 w/w) at 37° overnight. For the proteome preparation, we used the inStageTip (iST) method. Briefly, SDBRPS tips were washed with i) 100 µl acetonitrile (ACN), ii) 100 µl of 30% methanol and 1% TFA and iii) 150 µl of 0.2 % TFA centrifuging tips at 1000 xg for 3 minutes. Samples were loaded onto equilibrated columns and spin at 1000 xg for 10 minutes. SDBRPS tips were washed with i) 100 µl of 1% TFA, ii) 100 µl of 1% TFA in isopropanol and iii) 0.2% TFA. For the elution of proteins, we used a buffer containing 80% ACN, 5% NH_4_OH in MilliQ water. Samples were centrifuged at 1000 xg for 4 minutes and concentrated by SpeedVac at 45° for ~45 minutes. Finally, samples were dissolved in 10μl of a buffer containing 2% ACN and 0.1% TFA.

**Mass spectrometry analyses**

The peptides were desalted on StageTips and separated on a reverse phase column (50 cm, packed in-house with 1.9-mm C18- Reprosil-AQ Pur reversed-phase beads) (Dr Maisch GmbH) over 120 min or 140 min (single-run proteome and phosphoproteome analysis respectively). After elution, peptides were electrosprayed and analyzed by tandem mass spectrometry on a Orbitrap Exploris 480 (Thermo Fischer Scientific). The instrument was set to alternate between a full scan followed by multiple HCD based fragmentations scans for a total cycle time of up to 1 s.

**Proteome Data processing**

DIA Raw files were analyzed with Spectronauts HTRMS converter and analyzed with Spectronaut (v15.7.220308.50606). MS/MS spectra were matched against the *Mus musculus* UniProtKB FASTA database (September 2014), with an FDR of < 1% at the level of proteins, peptides and modifications. Enzyme specificity was set to trypsin, allowing for cleavage N-terminal to proline and between aspartic acid and proline. The search included cysteine carbamidomethylation as a fixed modification. Variable modifications were set to N-terminal protein acetylation and oxidation of methionine. Where possible, the identity of peptides present but not sequenced in a given run was obtained by transferring identifications across liquid chromatography (LC)-MS runs (‘match between runs’). Peptides had to be fully tryptic and up to two missed cleavages were allowed for protease digestion.

**Protein Correlation Profiling Analysis**

Spatial proteomics data were analyzed using Perseus (version 1.6.15.0) following the strategy reported in Figure 1. Briefly, label-free quantification (LFQ) intensities obtained from MaxQuant were used as input. The full dataset was first split into four independent matrices, one for each biological condition (JMD CTRL, JMD MIDO, TKD CTRL, and TKD MIDO). Median LFQ intensities across the four biological replicates were calculated for each subcellular fraction. The median intensities were scaled to a 0-1 range. Proteins not quantified in a given fraction were assigned an intensity value of 0. Reproducibility was evaluated by calculating Pearson correlation coefficients between biological replicates for each fraction. Marker lists were derived from previously published and curated datasets Krahmer et al., 2018. We manually curated this list to adapt it to our experimental framework: specifically, we categorized all membrane-bound compartments (e.g., ER, Golgi apparatus, mitochondria, etc.) under the broader category “organelles” and excluded categories not compatible with our fractionation strategy, such as protein complexes. Subcellular localization was inferred using a supervised support vector machine (SVM) classification approach implemented in Perseus. A radial basis function (RBF) kernel was applied, with parameters set to sigma = 0.2 and C = 8. The same model parameters were applied uniformly across all experimental conditions without further optimization. Learning was performed once per condition and not separately for individual biological replicates. Proteins were classified into three major subcellular compartments (cytosolic, nuclear, and organellar) based on their fractionation profiles. Classification performance was evaluated using the marker proteins, yielding high assignment accuracy (Fig. S2C). Proteins were retained for downstream analysis only if they showed a positive assignment to at least one compartment. Pearson correlation coefficients were used as similarity metrics for assessing reproducibility between biological replicates. Correlation values were not used as measures of statistical significance. Proteins were defined as having a localization change if organelle assignments in basal condition were different to that upon midostaurin treatment.


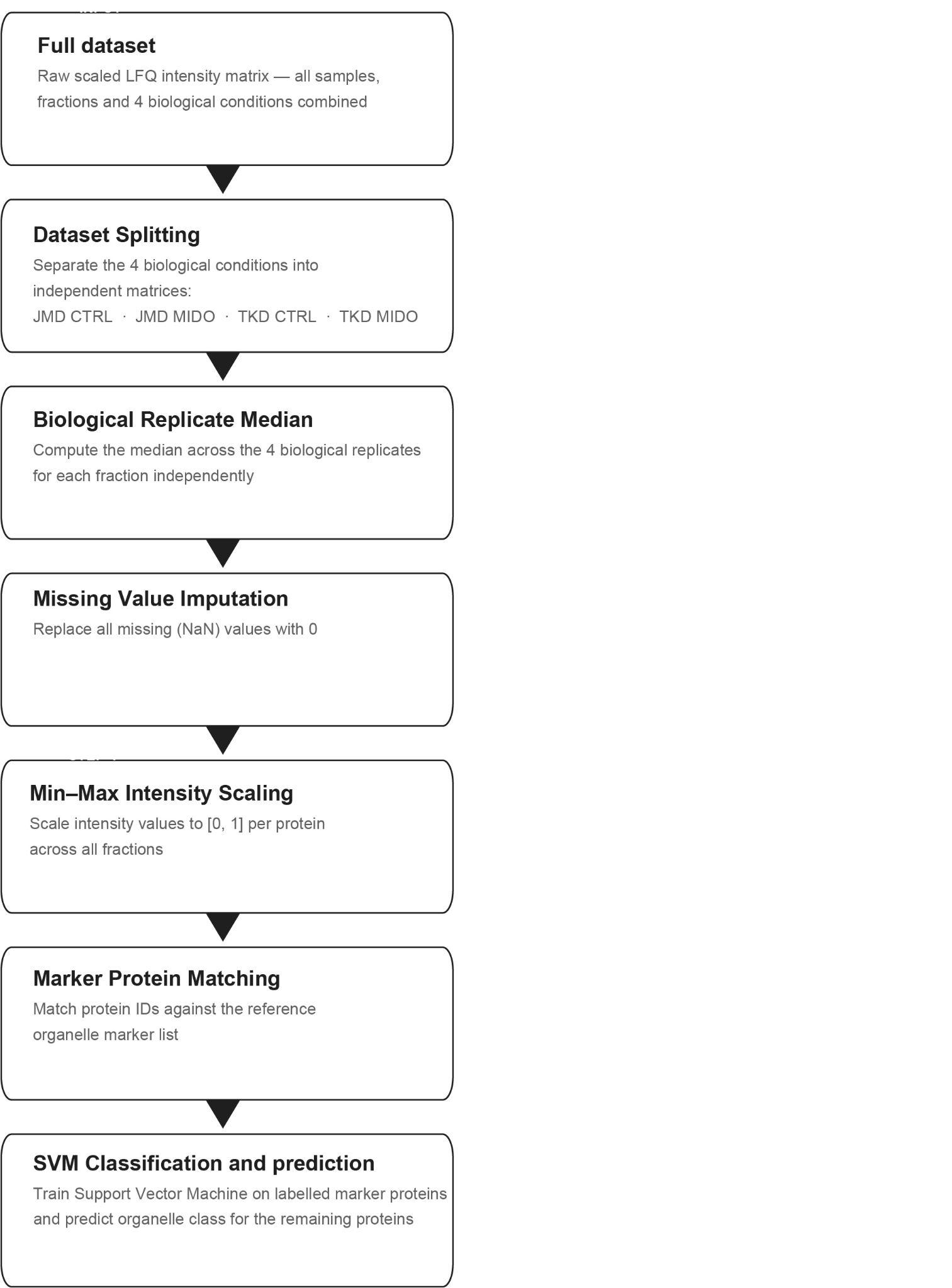


**Figure 1.** Strategy for Protein correlation profiling analsys.

**Statistical evaluation of organelle assignment and protein re-localization upon midostaurin treatment.**

For each protein and each experimental condition, we computed pairwise Pearson correlations between the sum-normalised fractionation profiles of all biological replicates (up to six pairwise comparisons for four replicates) (Table S9). The mean of these pairwise correlations constitutes the Localization Score for that protein: a value of 1 indicates that all replicates give identical profiles, while 0 indicates no agreement. For the proteins identified as relocalized, the median pairwise Pearson r was positive across all conditions, confirming that replicates consistently agree on the shape of each protein’s fractionation profile within a given condition. For each protein in each condition, we correlated the sum-normalised profile of every individual biological replicate against the mean profile computed across all replicates. A high replicate-vs-mean Pearson r confirms that the mean faithfully represents each replicate and that no replicate constitutes an outlier that would distort the SVM input (Table S9). To confirm that the identified relocalization events are reproducible across replicates, we computed the Euclidean distance between the sum-normalised CTRL and MIDO fractionation profiles independently for each biological replicate. A large Euclidean distance indicates a major redistribution of the protein across fractions between the two conditions. Proteins identified as relocalized showed significantly larger CTRL↔MIDO distances than proteins with stable localisation (Table S9).

**Seahorse analysis**

Bioenergetics of Ba/F3 cells were analyzed after prior 24h culture in absence or presence of 100 nM PKC412. Cells were analyzed with the Seahorse XF Glycolysis Stress Test (Agilent), the Seahorse XF Mitochondrial Stress Test (Agilent) and the Seahorse XF Mito Fuel Flex Test (Agilent) according to the manufacturer’s recommendations on a Seahorse XFe 96 (Agilent) with 100.000 cells per well.

**Electron microscopy**

Ba/F3 cells expressing FLT3^ITD-JMD^ and FLT3^ITD-TKD^ constructs were fixed with 1% glutaraldehyde in 0.2 M HEPES buffer (pH 7.3) for 30 min at room temperature. Samples were washed three times with 1× PBS and centrifuged in the presence of 1% BSA for 10 min at 13,200 rpm until a compact cell pellet was obtained. Cell pellets were post-fixed for 30 min on ice with a mixture of 2% osmium tetroxide (OsO₄) and 3% potassium ferrocyanide. Subsequently, samples were incubated with 1% thiocarbohydrazide (TCH) diluted in H₂O for 5 min at room temperature. Pellets were then incubated overnight at 4 °C with 0.5% uranyl acetate diluted in ddH₂O. The following day, samples were dehydrated through a graded ethanol series (50%, 70%, 90%, and 100%) for 10 min at each step. Dehydrated pellets were infiltrated with a 1:1 mixture of acetone and EPON resin for 2 h, followed by incubation in pure EPON resin for an additional 2 h. Finally, samples were polymerized at 60 °C for 48 h. Three washes with ddH₂O were performed between each processing step. Serial ultramicrotomy was performed using a Leica EM UC7 ultramicrotome (Leica Microsystems), and thin sections (60 nm) were collected onto formvar carbon-coated slot grids. Imaging was performed using a Tecnai-12 transmission electron microscope (FEI) equipped with a VELETTA CCD digital camera (Soft Imaging Systems). Quantitative analysis of mitochondrial ultrastructure was performed using Fiji (ImageJ) image analysis software. Total mitochondrial area, the number of cristae per mitochondrion, and the cristae area normalized to the corresponding mitochondrial area were measured.

**Flow cytometry analysis of FLT3 protein level**

Flow cytometry was performed to assess surface and total protein expression. Cells were harvested into pre-labeled FACS tubes and washed once with phosphate-buffered saline (PBS). To discriminate live from dead cells, 2 µL of GhostDye Red 780 (diluted 1:100) was added to each sample and incubated for 15 minutes at room temperature in the dark, followed by one wash with PBS. 10 µL of Gamunex were added in each sample and incubating for 10 minutes at 4°C. Each sample was then divided into two aliquots for parallel surface and total protein staining. For surface staining, 5 µL of AF647-conjugated anti-CD135 was added and incubated for 20 minutes at 4°C, followed by one wash with PBA. For total protein staining, cells were fixed and permeabilised by adding 250 µL of BD Cytofix/Cytoperm solution and incubating for 30 minutes at 4°C. Cells were then washed twice with 1x BD Perm/Wash buffer, blocked with 10 µL of Gamunex for 10 minutes at 4°C, and stained with 5 µL of AF647-conjugated anti-CD135 for 30 minutes at 4°C, followed by one final wash with 1x BD Perm/Wash buffer. Reference controls and all samples were acquired on a Cytek spectral flow cytometer.

**Statistics**

All the experiments have been conducted in at least 3 independent replicates obtained from 3 cell line batches (n = 3). Data are presented as means ± standard error of the mean (SEM). Multiple comparisons between three or more groups were performed using one-way or two-way ANOVA. Statistical significance between two groups was estimated using the unpaired t test assuming a two-tailed distribution. Statistical significance is defined as *p < 0.05; **p < 0.01; ***p < 0.001. All statistical analyses were performed using Prism 7 (GraphPad).
